# “Malignant” Perivascular Epithelioid Cell Neoplasm: Risk Stratification and Treatment Strategies

**DOI:** 10.1155/2012/541626

**Published:** 2012-04-26

**Authors:** Jonathan S. Bleeker, J. Fernando Quevedo, Andrew L. Folpe

**Affiliations:** ^1^Division of Medical Oncology, Department of Oncology, Mayo Clinic, Rochester, MN 55905, USA; ^2^Department of Laboratory Medicine and Pathology, Mayo Clinic, Rochester, MN 55905, USA

## Abstract

*Purpose*. Perivascular epithelioid cell tumors (PEComas) are a rare collection of tumors characterized by a myomelanocytic phenotype, and PEComas occurring in “nonclassic” anatomic distributions are known as perivascular epithelioid cell tumor not otherwise specified (PEComa-NOS). This review aims to compile and analyze cases of PEComa-NOS in an effort to better define their natural history. *Design*. We evaluated all 234 cases of PEComa-NOS reported in the English literature, extracting information regarding diagnostic features, treatment approaches, and outcomes. Multivariate analysis of a number of variables evaluable on pathologic review was performed to refine preexisting risk stratification criteria. Outcomes for patients receiving nonsurgical treatment are also reported. *Results*. Primary tumor size ≥5 cm (*P* = 0.02) and a high (1/50 HPF) mitotic rate (*P* < 0.0001) were the only factors significantly associated with recurrence following surgical resection. Cytotoxic chemotherapy and radiation therapy have shown little benefit in treating PEComa-NOS; mTOR inhibition is emerging as a treatment option. *Conclusion*. Progress has been made in understanding the natural history and molecular biology of PEComa-NOS. This review further clarifies risk of recurrence in this disease, allowing clinicians to better risk stratify patients. Further work should focus on applying this knowledge to making treatment decisions for patients with this disease.

## 1. Introduction

Perivascular epithelioid cell tumors (PEComas) are a collection of rare tumors defined by the World Health Organization as “mesenchymal tumors composed of histologically and immunohistochemically distinctive perivascular epithelioid cells” [1]. In 1991, Pea et al. described the presence of a unique cell with “prominent cytoplasmic borders and clear to granular, eosinophilic cytoplasm” in a perivascular distribution in both angiomyolipoma (AML) of the kidney and clear cell sugar tumor (CCST) of the lung [2]. The same group coined the term “perivascular epithelioid cell” (PEC) for these cells in 1992 and proposed the presence of this distinctive cell, distinguished in part by strong HMB-45 positivity, as a common link between a number of rare disorders in disparate locations, including AML, CCST, and lymphangiomyomatosis (LAM) of the lung [3]. Despite much controversy regarding the significance of PECs [4], including the fact that no normal counterpart of the PEC is known, further descriptions of tumors in multiple anatomic locations composed primarily of these cells has led to them being designated “PEComas” [5]. PEComas are notable for a myomelanocytic phenotype, with nearly all being immunoreactive for both melanocytic (HMB-45 and/or melan-A) and smooth muscle (actin and/or desmin) markers. The PEComa family of tumors is now felt to be comprised of AML, CCST, LAM, and less well-characterized PEComas of a variety of other anatomic origins, for which the term “perivascular epithelioid cell tumor not otherwise specified” (PEComa-NOS) has been proposed [6].

PEComa-NOS have been described in a variety of anatomic locations, including the colon [8, 9], pancreas [5, 10], retroperitoneum [11, 12], heart [13, 14], adrenal gland [15], breast [16], eye [17], biliary tract [18], bone [19], urinary bladder [20, 21], skull base [22], liver [23–25], uterus [26], cervix [27], skin [28], nasopharynx [29, 30], upper airway [31], and soft tissues [32, 33]. The majority of the published literature regarding PEComa-NOS is in the form of case reports and series, with a focus on the presentation and distinguishing pathologic features of the disease. In these reports, PEComa-NOS has been given a variety of titles, including “clear cell sugar tumor,” “primary extrapulmonary sugar tumor,” “clear cell myomelanocytic tumor,” and “monotypic epithelioid angiomyolipoma.” This review aims to compile data from the available literature on PEComa-NOS with a focus on risk stratification and treatment strategies which have been employed in various stages of the disease. Given improving recognition of PEComa-NOS and an increasing number of reported cases, this review aims to build on previously established risk stratification criteria to improve management of patients with this rare disorder.

## 2. Materials and Methods

### 2.1. Literature Review

Cases included in this analysis were retrieved via search of the PubMed database (United States Library of Medicine) for English-language publications prior to December 1, 2010. Search terms included “PEComa,” “perivascular epithelioid cell,” “perivascular epithelioid cell tumor,” “clear cell sugar tumor,” “primary extrapulmonary sugar tumor,” “clear cell myomelanocytic tumor,” and “monotypic epithelioid angiomyolipoma.” Cases of AML, LAM, and CCST occurring in their typical anatomic location were not included in this analysis. Cases described as “monotypic epithelioid angiomyolipomas” which occurred outside the kidney were considered PEComa-NOS; “typical” AML cases not having monotypic epithelioid features were not included in this analysis regardless of anatomic location. Cases which were not considered PEComa-NOS by the primary authors, but which have subsequently been included in case series based on pathologic findings [4], were also included in this analysis. One difficulty in defining the natural history of PEComa-NOS is the large number of patients who had undergone resection of what was felt to be a different entity a number of years earlier and subsequently presented with PEComa. Unless the initial pathology was reviewed and felt to be consistent with primary PEComa-NOS, cases such as this were considered as new cases and not evidence of recurrence.

### 2.2. Statistical Analysis

A total of 234 cases in a total of 116 reports were retrieved using the above criteria and reviewed to obtain demographic information, pathologic details regarding the primary tumor and outcome data. Statistical evaluation for risk of recurrence after surgical resection was performed utilizing JMP v. 9.0.1 (Cary, NC). Univariate analysis was performed using the Cox proportional hazards method. Variables deemed significant were then analyzed for correlation; when two variables were highly correlated, the variable with the most significant *P*-value was included in the multivariate analysis, which was performed using forward stepwise multiple logistic regression. Multivariate analysis was performed utilized all noncorrelated variables; given the large number of cases with missing data points, multivariate analysis was deemed valid only if there were at least 10 events for each variable included in the Cox proportional hazard analysis. The level of significance was set at *P* < 0.05.

## 3. Results

### 3.1. Review of Reported Cases

Patients ranged in age from 3 to 97 years (median 43 years) and 79% of patients were female (186/234). When gender-specific locations (prostate, uterus) were excluded, the strong female predominance persisted, with 73% (125/172) of cases occurring in females. The uterus was the most common anatomic site of origin, comprising 20% (47) of cases. Other common sites of origin included the skin (22 cases), liver/falciform ligament (20 cases), retroperitoneum (18 cases), and colon/rectum (16 cases). Tumor size in cases where such information was reported ranged from 0.5–30 cm (mean 6.8 cm). Lymph node involvement was noted in 5 cases, and distant metastatic disease was present at diagnosis in 6.8% (16/234) of cases. Local disease was deemed unresectable in 2 additional cases. The most common location for distant metastatic disease at the time of diagnosis was the lung (5 cases) followed by bone (4 cases), with ovarian, liver, adrenal, and peritoneal metastatic disease also reported.

Some form of followup was available in 81% (189/234) of cases, with the duration of followup ranging from 1 month to >15 years. Information regarding treatment was available in 95% of cases (222/234); the degree of followup and treatment information varied widely. A total of 20 patients were dead of disease at the time of reporting, comprising 10.6% of cases with followup information. Of these 20, 7 patients presented with metastatic or unresectable disease and the other 13 had recurrence after surgical resection.

### 3.2. Risk Stratification and Outcomes

Folpe et al. [7] identified a number of “high-risk” histopathologic features and integrated them into a set of criteria to risk stratify PEComas into “malignant,” “uncertain malignant potential,” and “benign” categories (Table 1). All cases included in this review were analyzed for presence of these factors and risk stratified based on these criteria. Given the fact that not all cases contained complete reporting on all these high-risk features, risk status was only able to be conclusively determined in 40% (93/234) of cases. The large majority of cases which could be definitively stratified using the criteria (87/93; 94%) were classified as “malignant,” 6 were classified as “benign,” and none were definitively classified as “uncertain malignant potential,” although 30 cases with partial reporting of high-risk features met at least this level of risk based on size alone. Of the 87 cases deemed “malignant,” 14 had evidence of distant metastases or unresectable disease at diagnosis. Surgical resection of metastatic disease was felt to be complete in 3 of these 14 cases in addition to 68 cases without metastatic disease where the disease was felt to be completely surgically resected.

In cases with reported followup, a total of 56 cases (29.6%; 56/189) showed evidence of “malignant behavior,” defined as metastatic or unresectable disease at diagnosis or recurrence after initial surgical resection. Eighteen of these 56 cases presented with metastatic/unresectable disease and 38 had recurrence following what was felt to be complete initial surgical resection. Of the 38 cases of recurrence, 31 were able to be fully categorized using the Folpe criteria, and all 31 fell into the “malignant” subgroup. No cases of malignant behavior were noted in patients in the “benign” or “uncertain malignant behavior” subgroups, although risk stratification was, as mentioned, incomplete for 7 cases. Only 2 cases of recurrence occurred in patients with a primary tumor <5 cm, and both of these cases demonstrated at least one other high risk feature. The rate of “aggressive behavior,” defined as metastatic or recurrent disease, in cases with followup classified as “malignant” by the Folpe criteria was 51% (39/76), less than the 71% originally reported by Folpe et al. [7].

In an effort to build on the Folpe criteria for risk stratification focusing on risk of recurrence after what was felt to be complete surgical resection, Cox proportional hazard testing was performed for a number of variables, including those comprising the Folpe criteria. Results of the univariate analysis are shown in Table 2. Essentially, all factors except infiltration included in the Folpe criteria were significantly associated with an increased risk of recurrence after surgical resection. Increasing age was also significantly associated with recurrence, and cutaneous primary tumors were associated with a lower recurrence rate; in fact, no cases of cutaneous PEComa-NOS recurred after surgical resection.

All variables significant in the univariate analysis were then analyzed for correlation in terms of impact on risk of recurrence. Factors that were significantly correlated included high mitotic rate and high grade (Grade 3); presence of vascular invasion and necrosis were also significantly correlated. Given these correlations, only the correlated variable with the most significant *P*-value on univariate analysis (high mitotic rate, necrosis) was included in the multivariate analysis, which was performed utilizing a stepwise multiple logistic regression. The results of the multivariate analysis are presented in Table 3. Only size >5 cm and high mitotic rate were significantly associated with recurrence, although the number of events present in the analysis was too small to consider this a valid estimate of risk. Thus, these two variables were analyzed separately, and they both retained a significant association with recurrence after surgical resection: size ≥5 cm (hazard ratio: 4.30, 95% CI 1.23–27.14, *P* = 0.02) and high mitotic rate (hazard ratio: 7.56, 95% CI 2.97–23.19, *P* < 0.001). All other variables included in the initial multivariate analysis were analyzed sequentially with these two factors; in no analysis were any of the other factors significantly associated with recurrence or did the two significant risk factors lose their association.

Utilizing these two significant risk factors for recurrence, a revised set of risk stratification criteria were applied to the cases reviewed, with cases with a primary tumor <5 cm in size and without a high mitotic rate defined as “benign,” those with one of these features as “uncertain malignant potential,” and those with a primary tumor ≥5 cm in size and a high mitotic rate as “malignant.” These criteria were compared with the previously described Folpe criteria to determine predictive accuracy in this large series of cases. Only cases where all components of the applied risk stratification criteria were available were included in the analysis in an effort to avoid underestimating risk of recurrence in patients missing data; the results of this analysis are in Table 4.

### 3.3. Treatment Approaches

222/234 cases reported some details of treatment; of these, 216 underwent either excisional biopsy or surgical resection of the primary tumor. In cases where surgery was not attempted, the disease was metastatic at presentation or the primary lesion was felt to be unresectable. A minority of patients (41/234; 18%) underwent therapy in addition to surgery, as summarized in Table 5.

#### 3.3.1. Neoadjuvant Therapy

Six patients underwent neoadjuvant therapy; 3 were treated with neoadjuvant chemotherapy alone, 2 with radiation alone, and 1 with chemotherapy followed by radiation. The clinical and pathologic response to neoadjuvant therapy in these cases was mixed. Jeon et al. [34] described a uterine PEComa in a 9-year-old girl treated with 2 cycles of neoadjuvant vincristine, ifosfamide, and doxorubicin leading to a 10% reduction in tumor size on imaging. She subsequently underwent surgical resection; no significant treatment effect was noted on the pathology specimen. This was followed by concurrent chemoradiation resulting in no evidence of disease 17 months following resection, although she did develop acute lymphoblastic leukemia felt to be secondary to exposure to anthracyclines [35]. The most robust response to neoadjuvant therapy was described by Osei et al.[37], where 6 cycles of doxorubicin and ifosfamide led to an 80% decrease in the size of an upper extremity soft tissue PEComa. This was followed by neoadjuvant radiation, during which the tumor progressed. At the time of resection, the tumor showed 20% necrosis. In other cases utilizing neoadjuvant chemotherapy alone, progression on therapy [36] and presence of residual viable tumor [7] have been noted. In cases where neoadjuvant radiotherapy was utilized [38, 39], little clinical or pathologic information was provided to attest to efficacy. Five of these 6 cases were able to be classified as “malignant” using the Folpe criteria. The one patient who suffered recurrence was the only patient to have a significant response to neoadjuvant therapy, as reported by Osei et al. [37].

#### 3.3.2. Adjuvant Therapy

A total of 19 patients received adjuvant therapy, with 7 receiving systemic chemotherapy, 8 undergoing radiotherapy, and 2 receiving concurrent chemoradiation; hormonal therapy (tamoxifen) and immunotherapy (Interferon-alpha) were both utilized in one case. Information was available to risk stratify 12 of these 19 cases; all 12 were “malignant” utilizing the Folpe criteria. The other cases were unable to be definitively classified, but 3 of the seven were at least of “uncertain malignant potential” due to size alone.

Information regarding adjuvant chemotherapy regimens was available in 5 reports; the majority of regimens utilized an anthracycline backbone. Followup was available for 6 of the 7 patients receiving adjuvant chemotherapy alone; 5 of these 6 patients had recurrent disease at a median of 21 months of followup (range 2–36 months). Three of these patients were dead at the time of reporting; two of 8 patients receiving adjuvant radiotherapy experienced recurrence.

#### 3.3.3. Recurrent/Metastatic Disease

As mentioned above, 38 patients with reported followup had evidence of recurrence; time to discovery of recurrence ranged from 1–180 months (median 23 months). Recurrence patterns were diverse, but the most common sites of recurrence were local (15 cases) and in the lungs (15 cases). Liver, CNS, and bone metastases were also frequent, with single reports of metastasis to the pancreas [55], heart [52], and bladder [55].

Surgical resection of isolated metastatic disease has been an effective approach in some cases, as a number of patients were rendered disease-free with surgical resection alone [56, 57]; multiple patients required serial resections for recurrent metastases [39, 55]. Only 7 patients received nonsurgical therapy following recurrence, with 3 receiving chemotherapy (paclitaxel [51], carboplatin, and epirubicin [11]), 1 undergoing radiation to a bony metastasis [49], and 3 recently reported cases treated with an mTOR inhibitor [52, 54]. Only one of these patients was deceased at the time of reporting; the rest were alive with disease.

A total of 18 patients presented with either metastatic disease not amenable to resection (16 cases) or unresectable disease without evidence of distant metastases (2 cases). Of these 18 patients, 10 underwent nonsurgical therapy, with 3 receiving chemotherapy, 5 undergoing combined chemoradiation, and 2 receiving an mTOR inhibitor. Followup was available for 13 of these 18 patients; with 7 of the 13 dead of disease at a median of 16 months (range 4–30 months).

## 4. Discussion

### 4.1. Clinical Features

The results of this review largely corroborate the results of other large case series investigating PEComas regarding the clinical features of the disease. PEComas can occur at the extremes of age, but the median age of 43 years in this review is consistent with prior series. A strong female predominance has been a consistent finding in PEComa series, and this review confirms that finding, although the approximately 4 : 1 female-to-male ratio is slightly less than that noted in earlier series [7, 58] One potential reason for this is an increase in the number of extragynecologic PEComas being recognized, as only 24% (56) of the cases reviewed here were gynecologic in origin as compared to approximately 40% in earlier series [7, 59]. Increased recognition of the PEComa family of tumors has likely led to improved characterization of these tumors in unusual locations, whereas they may have been mischaracterized as melanoma or soft tissue sarcoma in the past.

### 4.2. Risk Stratification

Folpe et al. [7] established criteria for determining malignant potential of PEComas in 2005 (Table 1). These criteria have been applied in multiple subsequently reported cases, although the role of these criteria in guiding management remains unclear. Information to allow for complete application of these criteria was available in only 40% of reviewed cases, making drawing conclusions from this data difficult. However, the available data do allow for further evaluation of the criteria in predicting recurrence following what is felt to be complete resection of a PEComa-NOS. Of the 38 cases of recurrence in this review, 31 (82%) were classified as malignant using these criteria. Information required for complete risk stratification was incomplete in the remaining seven patients with reported recurrence, but 3 of these 7 cases were categorized as having “uncertain malignant potential” due to size ≥5 cm alone. With full reporting of all high-risk criteria, these cases may have been recategorized into the “malignant” group. No documented recurrence occurred in any patient with a primary tumor <5 cm in greatest diameter without an additional high risk feature. These findings provide support for the benign nature of PEComa-NOS which are <5 cm in size without any other high-risk features as defined by the Folpe criteria.

In evaluating the multivariate analysis and revised risk stratification criteria utilizing only size ≥5 cm and high mitotic rate as contributing risk factors, it is difficult to draw significant conclusions given the lack of reporting of these variables in a number of cases. In evaluable patients, presence of both of these factors predicted a higher risk of recurrence following surgical resection, while the absence of either of these high risk features appears to be associated with a very small risk of recurrence, and thus utilizing these two factors as the primary determinants of risk may simplify the risk stratification and counseling process in the postoperative setting. In practical terms, no risk stratification schema has been uniformly applied to guide decisions regarding adjuvant therapy, and continuing work will need to be done evaluating all potential adverse risk factors reported here as well as others yet to be described. Uniform reporting of all histologic features utilized in the Folpe criteria will no doubt be beneficial in efforts to further clarify such risk stratification criteria, as only 40% patients in this review were truly evaluable given incomplete reporting of these variables. Given this fact, applicability of the risk stratification we propose based on this data is limited; future series with more complete reporting will allow for improved statistical analysis and may lead to a different set of risk stratification criteria.

### 4.3. Treatment Approaches

This review demonstrates the wide variety of treatment approaches utilized in PEComa-NOS, as well as the lack of consensus regarding treatment strategies. No treatment approach has been shown to be consistently effective in this disease, although such a conclusion is certainly premature given the numbers of patients who have received treatment and the lack of randomized data available. One significant issue in the nonsurgical treatment of PEComa is that many cases are not diagnosed conclusively as PEComa until after surgical resection, limiting available treatment approaches.

Neoadjuvant treatment has been utilized in a small number of cases with only one report of a robust objective response to therapy; other cases utilizing neoadjuvant therapy report either progression or minimal evidence of efficacy in the resected tumor. The role of adjuvant therapy for PEComa-NOS is also unclear, and this series does little to change that. When reported, chemotherapy regimens utilized were typically similar to those used in soft tissue sarcomas with an anthracycline backbone, but many different variations were reported. The rate of recurrence (45%) and death from disease (15%) are much higher in this group than the series as a whole. This phenomenon has been noted in at least one other review of gynecologic PEComas [60], where receipt of postoperative therapy was associated with an increased rate of recurrence. No deaths appeared to be directly attributable to therapy, and it is most likely that the increased rates of recurrence and death in this group are due to selection of patients with extremely high-risk disease for adjuvant therapy as opposed to intrinsic harm from the treatment.

In the metastatic setting, systemic chemotherapy has shown little efficacy, as there were no objective responses reported with the use of chemotherapy in this review. The unpredictability of the natural history of PEComa-NOS, even in the metastatic setting, is also illustrated by the number of patients rendered NED by surgical resection of oligometastatic disease as well as reports of survival for up to one year without progression of disease in the absence of any therapy [61].

Targeted therapies, most notably mTOR inhibitors, have been a recent development in the treatment strategy of all members of the PEComa family, including PEComa-NOS. This interest initially arose from the observation that patients with tuberous sclerosis complex (TSC) have a higher rate of LAM and AML than the general population [62, 63]. TSC is an autosomal dominant genetic disease due to loss of either the *TSC1* (9p34) or *TSC2* (16p13.3) genes; a number of different mutations in either of these genes leads to the TSC phenotype [64, 65]. LAM is present in 25–35% of female patients with TSC [62, 66], 50–80% of adults with TSC have renal AMLs [63, 67, 68], and the two diseases are frequently both present in those with TSC [63]. Given this link between TSC, LAM, and AML, the role of *TSC1/TSC2 *mutations in the pathogenesis of these diseases has been vigorously investigated [69].

Germline loss of heterozygosity (LOH) at the *TSC2 *locus has been demonstrated in TSC-associated AML [70–72] and LAM [73]; LOH at the *TSC1 *locus has been less frequently described. *TSC1 *and *TSC2 *code for separate proteins which, after interaction, regulate cell proliferation via the mTOR pathway [74]. Complete loss of either *TSC1 *or *TSC2* due to a second hit to either *TSC* locus leads to a dysfunctional TSC1-TSC2 complex and unchecked cell proliferation through unregulated mammalian target of rapamycin complex 1 (mTORC1) activation [75] and impaired mammalian target of rapamycin complex 2 (mTORC2) activation [76]. Given the role of the mTOR pathway in these disorders, mTOR inhibition has been viewed with great enthusiasm as a potential treatment modality, and this enthusiasm has been matched by meaningful responses to mTOR inhibition in both AML and LAM [41, 77, 78].

The proportion of patients with PEComa-NOS and TSC is not as large as that linking TSC and LAM or AML [7], but genetic analysis of multiple PEComa-NOS specimens have demonstrated activation of the mTOR pathway [79, 80], indicating potential benefit for mTOR inhibition in PEComa-NOS. To date, the reported experience utilizing mTOR inhibition in PEComa-NOS is limited to a handful of case reports and series [46, 52, 54]. These series, however, have been promising in that significant responses [52], including at least one long-term response (16 months)[54], have been reported. Responses have not been uniform, however [46], and further clinical trial data must be accumulated before defining the role of mTOR inhibition in metastatic or recurrent PEComa-NOS. Clinical trials evaluating mTOR inhibitors in LAM and AML are currently accruing; it is unlikely given their rarity that PEComa-NOS treatment will be evaluated separately in a randomized fashion, so use of mTOR inhibitors in this setting will likely be in the setting of Phase I trials and/or off-label based on results of the above-mentioned trials in AML and LAM.

Given the available data in the literature, no definitive treatment strategy can be unequivocally recommended at this time. In the opinion of the authors, neoadjuvant therapy has its only role with a goal of conversion of unresectable disease to resectable, although little data supports the efficacy of such an approach. Adjuvant therapy may be of benefit in patients at high risk of recurrence, but given only approximately half of patients with disease classified as malignant by Folpe criteria will experience recurrence following resection, exposing patients to the risk of systemic chemotherapy without a known benefit is a major concern. Regardless of the postoperative strategy employed, long-term surveillance should be at its core, as multiple recurrences have been reported >5 years after surgical resection [52, 57, 81, 82]. Early detection of recurrence may allow for surgical resection of solitary lesions or oligometastatic disease, which has been effective in long term control of disease in a number of reported cases. Given the lack of benefit of traditional cytotoxic therapy in the metastatic setting, mTOR inhibition in the setting of a clinical trial should be strongly considered in any patient with recurrent or metastatic disease. This approach is as yet unproven in the adjuvant setting or following resection of metastatic disease, but mTOR inhibition is emerging as an attractive treatment option as more information on its use in LAM and AML returns.

## 5. Conclusion

PEComa-NOS remains a rare, but increasingly recognized entity and much progress has been made in unraveling the molecular mechanisms underlying this disease and other members of the PEComa family. Evaluation of risk of aggressive behavior remains an imprecise undertaking given the diverse array of clinical behavior noted in the literature, but this analysis suggests that tumor size and high mitotic rate are the best predictors of recurrence after surgical resection. The Folpe criteria continue to have utility, especially in helping to categorize lesions as having low malignant potential. This review better defines the natural history of PEComa-NOS of various primary sites and outlines the importance of long-term surveillance, given a number of cases of late recurrence. Ideal treatment strategies remain undefined, but an emerging role for mTOR inhibitors raises enthusiasm in the treatment of these rare tumors. Clinicians should be aware of this new treatment paradigm and work to ensure clinical trials to further define the role of mTOR inhibitors are embraced.

## Figures and Tables

**Table 1:** Proposed classification of PEComas (adapted from Folpe et al. [7]).

High risk features	Risk category
(1) Size >5 cm (2) Infiltrative growth pattern (3) High nuclear grade and cellularity (4) Mitotic Rate >1/50 HPF (5) Necrosis (6) Vascular invasion	“*Benign*” <2 high risk features and size < 5 cm****

	“*Uncertain malignant potential*” Size ≥5 cm with no other high risk features OR nuclear pleomorphism/multinucleated giant cells only****

	“*Malignant*” 2 or more high risk features

**Table 2:** Univariate analysis of clinical and histologic factors related to PEComa-NOS recurrence.

Variable	Recurrence rate	Risk ratio	95% CI	*P*-value
Age (continuous variable)		**1.02**	**1.01–1.04**	**0.01**
*Size *				
< 5 cm	6/92 (6.5%)	1		
≥ 5 cm	**32/84 (38.1%)**	**10.74**	**3.22–66.47**	**<0.001**
*Sex*				
Male	10/37 (27.0%)	1		
Female	28/134 (20.9%)	0.85	0.39–2.13	0.70
*Mitotic rate*				
Low	7/93 (7.5%)	1		
High (>1/50 HPF)	**24/44 (54.5%)**	**10.0**	**4.00–30.30**	**<0.001**
*Grade*				
Grade 1-2	9/51 (17.6%)	1		
Grade 3	**8/19 (42.1%)**	**3.35**	**1.17–9.42**	**0.03**
*Necrosis*				
Absent	8/79 (10.1%)	1		
Present	**25/53 (47.2%)**	**7.19**	**3.18–18.38**	**<0.001**
*Vascular invasion*				
Absent	11/52 (21.2%)	1		
Present	**9/17 (52.9%)**	**3.22**	**1.19–8.49**	**0.02**
Infiltration				
Absent	5/24 (20.8%)	1		
Present	7/38 (18.4%)	1.14	0.34–4.41	0.84
*Primary location* (versus all other sites)				
GYN	12/42 (28.6%)	1.37	0.62–2.83	0.42
GI	4/22 (18.2%)	1.69	0.49–4.51	0.24
Skin	**0/20 (0%)**	**6.2 × 10-7**	**NC**	**0.002**
Liver/Falciform ligament	2/18 (11.1%)	0.34	0.05–1.14	0.09
Retroperitoneal	4/12 (33.3%)	1.32	0.39–3.40	0.61

**Table 3:** Multivariate analysis of clinical and histologic factors related to PEComa-NOS recurrence.

	Variable	Risk ratio	95% CI	*P*-value
(a) All nonconcordant variables	Age	1.91	0.15–21.5	0.61
	Skin primary	0.0009	0-NC	0.93
Number of events: 24	Size >5 cm	**6.16**	**1.04–117.4**	**0.04**
Number of censorings: 83	High mitotic rate (>1/50 HPF)	**6.96**	**2.20–26.7**	**<0.01**
Total number: 107	Necrosis	1.55	0.56–4.70	0.41

(b) Variables significant on multivariate analysis	Size > 5cm	**4.30**	**1.23–27.2**	**0.02**
Number of events: 25	High mitotic rate (>1/50 HPF)	**7.57**	**2.96–23.2**	**<0.01**
Number of censorings: 93				
Total number: 118				

**Table 4:** Comparison of original and revised Folpe criteria in risk stratification.

Risk category	Recurrence following resection	
Benign	0/4 (0%)	1/46 (2.2%)
		
Uncertain		
Malignant	No cases	3/22 (13.6%)
Potential		
		
Malignant	31/68 (45.5%)	21/30 (70%)

**Table 5:** Reported cases of non-surgical treatment of PEComa-NOS.

Reference/year	Age/Sex	Location	Size (cm)	Folpe cat.	Management	Outcome	Treatment details
*Neoadjuvant cases*							
Folpe et al. [7]	49 F	Shoulder	N/A	Malignant	NA CT, resection	NED at 11 mo	Ifos/Adria—residual dz at resection
Jeon and Sung [34] and Jeon and Yi [35]	9 F	Uterus	6.5	Malignant	NA CT, resection, Adj CT	NED at 16 mo; ALL diagnosed at 16 mo	Vincr/Ifos/Adria—2 NA cycles, 6 adj cycles concurrent with 45 Gy.
Ong et al. [36]	8 F	Vagina	2	Unknown	NA CT, resection	NED at 6 mo	Ifos/Vincr/Actino D x3—prog
Osei et al. [37]	49 F	UE soft tissue	5.3	Malignant	Sequential NA CT then RT followed by resection	Single lung metastasis at 14 mo—resected	Ifos/Adria x6—80% reduction, then 50 Gy RT—prog
Weinreb et al. [38]	68 M	Thigh	7.8	Malignant	NA RT, resection	NED at 11 mo	
Yamashita et al. [39]	39 F	Tibia	6.5	Malignant	NA RT, resection	NED at 34 mo	

*Adjuvant Cases*							
Bosincu et al. [40]	48 F	Uterus	7	Malignant	Resection, Adj HT	NED at 36 mo	Tamoxifen
Chen et al. [41]	16 F	Abdominal	27	Malignant	Resection, Adj CT	Recurrence at 2 mo; no further follow-up	(+) LN at surgery
Fink et al. [42]	51 F	Broad Lig	17	Unknown	Resection, Adj RT	NED at 15 mo	50.4 Gy
Folpe et al. [43]	29 M	Falciform Lig	20	Malignant	Resection, Adj RT	Lung metastases at 3 mo; dead of other causes at 1 yr	
Folpe et al. [43]	10 F	Falciform Lig	5	Unknown	Resection, Adj CT	Lost to follow-up	Vincr, Actino D, Cyclophos
Folpe et al. [7]	71 M	Forearm	9	Malignant	Resection, Adj RT	NED at 10 mo	
Folpe et al. [7]	48 F	Cervix	2	Unknown	Resection, Adj RT	NED at 21 mo	
Folpe et al. [7]	77 F	Neck	2.6	Unknown	Resection, Adj RT	NED at 6 mo	
Folpe et al. [7]	56 F	Uterus	9	Malignant	Resection, Adj CT and RT	Lung and bony metastases at 11 mo; alive at 11 mo	
Folpe et al. [7]	59 F	Uterus	14.5	Malignant	Resection, Adj CT	Liver and lung metastases at 30 mo; alive at 30 mo	
Folpe et al. [7]	46 F	Mesentery	12	Malignant	Resection, Adj CT	Intra-abdominal and liver metastases at 22 mo; DOD at 27 mo	“Multiple” cycles of adjuvant CT
Folpe et al. [7]	36 F	Uterus	N/A	Malignant	Resection, Adj CT	Lung metastases at 12 mo, liver metastases at 36 mo; DOD at 39 mo	
Hornick and Fletcher [11]	50 F	Pelvis	13	Malignant	Resection, Adj RT	Lung, liver, abdominal wall metastases at 39 mo, alive at 46 mo	
Pan et al. [20]	46 M	Prostate	8.5	Malignant	Resection, Adj RT	Pulmonary metasases at 3 yrs, DOD at 4 yrs	Cis/Adria x6 cycles
Parfitt et al. [44]	48 M	Bladder	3	Unknown	Resection, Adj IT	NED at 48 mo	IFN-*α*
Ryan et al. [45]	15 F	Rectum	3.7	Malignant	Resection, Adj CT	NED at 9 mo	(+) LN at surgery; Ifos/Adria
Silva et al. [4]	76 F	Uterus	N/A	Malignant	Resection, Adj RT	NED at 8 mo	
Subbiah et al. [46]	58 F	Retroperitoneal	17	Unknown	Resection, Adj CT	Liver metastases at 2 yrs, resected with subsequent liver and lung metastases	2 cycles adj Adria; RT/adria after resection of liver recurrence; temsirolimus/bortezomib at 2nd recurrence—prog
Vang and Kempson [47]	75 F	Uterus	5	Unknown	Resection, Adj RT	NED at 30 mo	

*Metastatic/Recurrent Cases*							
Bonetti et al. [48]	19 F	Uterus	5.5	Malignant	Subtotal resection of primary, CT followed by RT	Local recurrence at 10 mo, diffuse metastatic disease at 18 mo	
Léon et al. [49]	76 F	Abdominal	15	Malignant	RT to sacral recurrence	Progressive metastatic disease	13 Gy RT to sacrum
Léon et al. [49]	38 F	Retroperitoneal	N/A	Malignant	CRT	DOD at 20 mo	RT/Thal
Fukunaga et al. [50]	40 F	Uterus	30	Malignant	Debulking surgery, CRT	DOD at 17 mo	Mulitple omental metastases at initial surgery
Greene et al. [51]	79 F	Uterus	13	Malignant	Debulking of pelvic recurrence, CT	DOD at “several” mo	Paclitaxel x1 cycle
Italiano et al. [52]	55 F	Uterus	N/A	Unknown	Resection of recurrence, CT, CRT following 2nd recurrence, mTOR following progression	Progressive metastatic disease; alive at 16 mo following recurrence	Ifos/Adria, RT+etop, Temsirolimus, Gem
Italiano et al. [52]	69 F	Uterus	N/A	Unknown	mTOR, resection of solitary lung metastasis	NED at 9 mo	Temsirolimus; response allowed surgical resection
Peng et al. [53]	47 M	Pelvis	12	Malignant	Subtotal resection of primary, CT	DOD at 9 mo	Ifos/Epi x2—prog; Adria/Oxali/Thal x 2—prog; Nada, Dacarb x2—prog
Silva et al. [4]	47 F	Uterus	N/A	Malignant	Resection of primary; RT followed by CT	DOD at 30 mo	Lung metastases at diagnosis; Adria/Cis x8 cycles
Silva et al. [4]	73 F	Uterus	N/A	Malignant	Resection of primary; CT	DOD at 9 mo	Paclitaxel x6 cycles
Silva et al. [4]	43 F	Uterus	N/A	Malignant	Resection of primary; CT	Alive with progressive disease at 6 mo	Ifos/Adria x5 cycles
Wagner et al. [54]	70 M	Kidney	9	Unknown	TKI, mTOR inhibitor, resection of recurrence	40% reduction in size of isolated recurrence, NED after resection	Sunitinib, sirolimus
Wagner et al. [54]	65 M	Retroperitoneal	20	Malignant	Resection of recurrences, mTOR inhibitor	“Near complete dissapperance” of multifocal disease at 12 mo, alive at 16 mo following recurrence	Sirolimus
Wagner et al. [54]	61 F	Cervix	9	Malignant	mTOR inhibitor	Initial response, DOD at 8 mo	Sirolimus
Yamashita et al. [39]	35 M	Vertebral	1.8	Malignant	CRT	Bone metastases at 9 mo, alive at 12 mo	Pelvic mets—no tx details
Yamashita et al. [39]	42 F	Uterus	15	Malignant	Resection of humeral recurrence, CT	DOD at 10 mo with diffuse metastases	

Legend: NA: Neoadjuvant, Adj: Adjuvant, CT: Chemotherapy, RT: Radiation therapy, IT: Immunotherapy, HT: Hormonal therapy, NED: No evidence of disease, DOD: Dead of disease, Ifos: Ifosfamide, Adria: Doxorubicin, Vincr: Vincristine, Actino D: Actinomycin D, CTX: Cyclophosphamide, Cis: Cisplatin, Thal: Thalidomide, Epi: Epirubicin, Oxali: Oxaliplatin, Nada: Nadplatin, Dacarb: Dacarbazine, Etop: Etoposide, Gem: Gemcitabine, TKI: Tyrosine kinase inhibitor.
